# Standards and Ontologies for Functional Genomics: Towards Unified Ontologies for Biology and Biomedicine

**DOI:** 10.1002/cfg.249

**Published:** 2003-02

**Authors:** Midori A. Harris, Helen Parkinson

**Affiliations:** European Bioinformatics Institute EMBL Outstation Wellcome Trust Genome Campus Hinxton, Cambridge CB10 1SD UK


**Comparative and Functional Genomics**
*Comp Funct Genom* 2003; **4**: 116–120.
Published online in Wiley InterScience (www.interscience.wiley.com). **DOI:** 10.1002/cfg.249

**Feature**

### Conference Report: Standards and ontologies for functional genomics: towards unified ontologies for biology and biomedicine

Wellcome Trust Genome Campus, Hinxton, Cambridge, UK, 17–20 November 2002

Midori A. Harris and Helen Parkinson*
*European Bioinformatics Institute, EMBL Outstation, Wellcome Trust Genome Campus, Hinxton, Cambridge CB10 1SD, UK*

*Correspondence to:
Helen Parkinson, European Bioinformatics Institute, EMBL Outstation, Wellcome Trust Genome Campus, Hinxton, Cambridge CB10 1SD, UK.
E-mail: parkinson@ebi.ac.uk*

Received: 28 November 2002
Accepted: 2 December 2002

### Introduction

In recent years, whole genome analysis has become routine and systems biology and modelling of whole cells are becoming more common. Advances in experimental technology now permit expression analysis for tens of thousands of genes at a time, generating vast amounts of biological data, and the application of high-throughput technologies to proteomics makes the burden even heavier.

Databases and tools have become available to help biologists manage and interpret these large volumes of data, but databases alone are not sufficient to allow researchers to integrate large amounts, and different kinds, of information. The problem is that any given biological phenomenon can be described in many different ways; an example is the use of free text annotation in databases such as GenBank, which has resulted in inconsistent data representation [2]. One promising solution is to provide consistent annotation within databases by means of standard formats and ontologies. Standards development depends on the availability of suitable ontologies; both the MIAME standard and emerging proteomics standards recommend the use of ontologies wherever possible. Where such ontologies do not exist, communities are starting to build their own to support standards [5].

Although the exact meaning of the word 'ontology' is contentious, T.Gruber's definition of an ontology as a 'specification of a conceptualization' (**http://www-ksl.stanford.edu/kst/what-is-an-ontology.html**) is widely used. Ontologies are, at a minimum, sets of terms used in a specific domain, definitions for those terms and defined relationships between the terms; they can range from simple controlled vocabularies to structurally complex representations employing description logics [4]. In biology, the use of ontologies allows database annotation to be standardized, and makes sophisticated queries possible for humans and computers. The SOFG conference brought together about 120 biological domain experts, computer scientists and those with interests in related fields, such as natural language processing (NLP), to address the issues of developing, implementing and ultimately unifying ontologies. Presentations covered specific ontologies being developed for a

Copyright © 2003 John Wiley & Sons, Ltd.

wide range of biological domains as well as tools for constructing ontologies and ways that ontologies are being put to use in various databases. It should be noted that ontologies are frequently developed for use in a database context, and do not exist in a vacuum.

In the context of this review, we evaluate ontologies on their content and potential use to biologists, rather than on the basis of construction methods. One insight that quickly emerged is that a research community must be actively involved in developing and deploying any ontology or standard for its field, to ensure that the ontology is relevant and usable. The emergence of the Gene Ontology (GO) vocabularies [6,7] as a *de facto* standard for gene product annotation illustrates the value of involving biologists in all stages of the development and application of an ontology. Numerous model organism databases (MGI, FlyBase, SGD, TAIR, WormBase and others), as well as SWISS-PROT and LocusLink, provide GO annotations for their database objects. These groups adopted GO voluntarily and now make active contributions to its development. The benefits of widespread community use of the GO extend beyond the model organism databases that generate it. Use of GO facilitates sequence annotation and speeds up the analysis of large-scale functional genomics experiments, and an increasing number of applications make use of GO terms and gene product annotations, e.g. several applications combine GO annotations with microarray data to address queries such as, 'Which GO terms are over-represented in a given cluster?'

We can go further in the use of ontologies than simply annotating gene products to the GO. Consider the following example: 'a species $X$, of developmental stage $Y$, has been treated with compound $Z$' and the resulting data matrix stored in a database. If the data alone are stored with just a free text description, query capability is limited. In contrast, if ontologies are used to describe the species, compound and developmental stage, structured queries, such as 'what experiments use compound $Y$?' are possible, as compound $Y$ is described only once within the database, and the source ontology provides an unambiguous definition for $Y$. This is precisely the type of query that the standard for microarray data, minimum information about a microarray (MIAME), was developed to answer [1].

### Keynote presentations

**Lincoln Stein (Cold Spring Harbor Laboratory, USA)** speculated on a seemingly universal human urge to collect and classify objects, a tendency that finds expression in classification systems for biology, and which mirrors the urge of children to collect, classify and describe Pokemon characters. He provided some insights into the sociology of naming things, a right that all scientists believe that they have. Using pathology as an example, he illustrated how classification systems require revision because they are often technology-based, and as technology changes, so the resolving possibilities of the system change.

**Winston Hide's (SANBI, South Africa)** keynote presentation used a population genetics metaphor to describe the spread of bioinformatics technology through the developing world, beginning in South Africa and extending to other parts of Africa and South America. Hide emphasized the point that open source software was, and is, essential to the development of infrastructure for open health care in much of the world, before going on to describe his group's efforts to provide an open controlled vocabulary, known as eVOC, for consistently describing clone, EST and cDNA libraries used in high-throughput experiments.

**Ken Buetow (National Cancer Institute, USA)** provided an overview of the National Cancer Institute's efforts to unify data related to cancer coming from sources as diverse as epidemiology, clinical trials, cancer animal models and microarray data into a unified system, with ontologies mapped across these domains into a common ontological representation environment called caCORE (**http://ncicb.nci.nih.gov/NCICB/core**). The goal of these efforts is to support all those involved in cancer research and facilitate *in silico* research. The NCI has not limited itself to a single technology, providing access to its data via Java, SOAP, XML and other formats and protocols, thereby placing itself at the cutting edge of data provision for cancer research.

**Peter Karp (SRI International, USA)** described various aspects of the BioCyc family of databases (**http://www.biocyc.org/**). Each database provides a knowledge base about a particular organism and covers several biological datatypes, such as genes, enzymes and metabolic pathways. Karp also summarized the features of the Pathway Tools software

Copyright © 2003 John Wiley & Sons, Ltd.

used to build and maintain the BioCyc databases and the underlying ontologies used.

**Michael Ashburner [European Bioinformatics Institute (EBI), UK]** discussed the Global Open Biology Ontologies (GOBO) effort, which aims to apply the community-based approach used by GO to other areas of biology (**http://www.geneontology.org/doc/gobo.html**). Key requirements for inclusion in GOBO are that the ontology be freely available, and available in a standard format. One subject area in which the GO community is directly involved is that of nucleic acid sequence types, as described in **Suzanna Lewis's (University of California at Berkeley, USA)** talk on the Sequence Ontology (SO) project. The SO categorizes sequence features along three orthogonal axes: an 'is-a' hierarchy of classes and subclasses (e.g. transcript is a subclass of RNA, which in turn is a subclass of nucleic acid); a classification based on location ('is-on'; e.g. an exon is located on a transcript); and a classification based on 'defines' (e.g. a transcript region defines a primary transcript). Representatives from model organism databases, and the EnsEMBL and DAS (distributed annotation system) projects contribute to the SO effort.

**Alexa McCray (National Library of Medicine, USA)** described the National Library of Medicine's Unified Medical Language System (UMLS), which integrates the contents of about 60 different source vocabularies into common concepts. The integration algorithms are available from UMLS website (**http://www.nlm.nih.gov/research/umls/**), and it is clear that the UMLS offers a unique view on ontologies for biology gained from the mapping process. In a related talk, **Jane Lomax (EBI, UK)** talked about issues encountered when integrating the GO vocabularies into UMLS, e.g. GO terms have synonyms, which fall into two distinct classes, true synonyms and related terms. This distinction means that merging with UMLS, a system that has many precisely defined relationships, will mean a re-evaluation of which synonyms are defined in the GO. Thus, inclusion of an ontology in UMLS can drive the development of that ontology.

The session on ***Ontologies for Model Organisms*** covered a wide taxonomic range, beginning with two talks on the laboratory mouse. In the first presentation, **Martin Ringwald (Jackson Laboratory, USA)** described ontologies in use in the databases provided by the Mouse Genome Informatics collaboration, emphasizing the combination of anatomy terms with qualifiers to generate phenotype descriptors. **Duncan Davidson (MRC Human Genetics Unit, UK)** then demonstrated the Mouse Atlas, in which links are made between text entries in an anatomy ontology and three-dimensional models (**http://genex.hgu.mrc.ac.uk/**). This approach facilitates the modelling of gene expression in three-dimensional space and over time during development. These models provide a unique and beautiful view on development of mouse tissues.

**Leszek Vincent (University of Missouri, Columbia, USA)** presented the work of the Plant Ontology Consortium (POC), echoing the theme of community-based ontology development. The POC aims to provide ontologies with grounding in phylogenetics, using structures (anatomy plus morphology) in *Zea mays* as an initial test case. **Sue Rhee (Carnegie Institution of Washington, USA)** described several ontologies developed by the *Arabidopsis* Information Resource (TAIR), covering anatomy, developmental stages and phenotypes, and used in literature-based database curation (**http://www.arabidopsis.org/info/ontologies/**). The model organism session also encompassed the nematode *C. elegans* [3], the fruit fly *Drosophila* (**Huaiyu Mi**), and fungi (**Gregory Butler**).

The ***Implementation and Use of Ontologies*** session focused on how ontologies can be used within microarray databases [5] and in industrial settings. **Bo Servenius (AstraZeneca, Sweden)** described a joint project with Spotfire (vendors of microarray analysis software), in which AstraZeneca contributed annotation and definitions to the GO project and then incorporated these into the tools they use locally for microarray analysis. In another industry perspective, **Robin McEntire [GlaxoSmithKline (GSK), USA]** outlined GSK's approach to incorporating the efforts in ontology and standards development into their drug discovery pipelines. Robin emphasized the need for GSK to use public efforts and their willingness to contribute to them by unveiling a tissue type ontology, constructed in-house, which they intend to make public. In the same session, **Christian Blaschke (Universidad Autonoma Madrid, Spain)** gave an NLP perspective on the ontology world in a talk which described a method for mining terms from the literature, and applying these to classify poorly described gene products. This use of the data in

Copyright © 2003 John Wiley & Sons, Ltd.

the literature to generate structured knowledge was confirmed by use of the GO, which largely verified the results of the text mining.

**Diane Oliver (Stanford University, USA)** presented a knowledge base constructed for representing SNP data in the context of clinical outcomes after drug treatment. She provided examples of known SNPs that can affect patient survival in combination with drug treatment, and showed how three ontologies have been imported into a common environment created using the Protégé tool. This knowledge base is now being used to generate forms through which clinicians can submit clinical data, and represents a use of ontologies and knowledge representation that has a direct health care benefit. Protégé was described in detail by **Mark Musen (Stanford University, USA)** in the session ***Tools for Building Ontologies***. Protégé — in a familiar theme — is a tool that is under development by the community that use it, and Musen described a number of plug-ins that are available from the Protégé website (**http://protege.stanford.edu/**). **Sean Bechhofer (University of Manchester, UK)** complemented the description of Protégé with an explanation of how the standard format DAML + OIL evolved into OWL, a format also used by Protégé, and explained how OilEd, an ontology editor, works.

In the ***Ontologies for Chemistry, Toxicology and Other Domains*** session, both **Andrew Jones (University of Glasgow, UK)** and **Steve Oliver (University of Manchester, UK)** gave presentations on the need for proteomics standards. **Steve Oliver** introduced PEDRo, an initiative that provides a database model for proteomics data based on the maxD system of the University of Manchester, while **Andrew Jones** presented a modification of the microarray community data model for proteomics experiments. Interestingly, these approaches are complementary, and the meeting provided a forum for those interested in developing standards specifically for proteomics.

**Chris Catton (Oxford University, UK)** presented the BioImage project (**http://www.bioimage.org/**), highlighting the need for image archiving and illustrating the challenges involved in describing images. While image and video formats are relatively controlled, the subjects of the images can be interpreted in many ways. He provided an interesting example of how interpretation and metadata affect image interpretation: at first glance, an image of Jackie Kennedy wearing a leopard-skin coat looks like a simple news story, but it started a fashion which had a dramatic impact on the wild population of the animal in question.

This session was started with an introduction to the field of chemical ontologies. **Tony Davies (IUPAC, Germany)** provided a review of the structure of IUPAC and outlined the formal way in which they work to provide unique chemical identifiers. He was followed by **Kirill Degtyarenko (EBI, UK)** on the needs that biochemists have for specific types of chemical ontologies. The key areas that he identified were related to structure, enzymatic reactions and bioinorganic proteins. He also noted flaws in the EC system, which limit its use in describing certain enzyme activities.

**Mike Waters (National Institute for Environmental Health Sciences, USA)** outlined some of the ways that metadata is gathered in high-throughput toxicogenomics experiments. To aid in standardizing this data, standardized pathology tables are used, and will be included in a developing knowledge base called CEBS. He also outlined the problems in working with the rat, which has not historically been a genetic model organism, and described a mapping project in which emerging rat genomic and EST data are reannotated based on information in the literature. This resource provides detailed annotation with a toxicogenomic slant, which is currently not available in public sequence databases.

### Conclusions

The importance of involving the research community in developing standards and ontologies was highlighted by many of the speakers; together with the development and use of open standards and open source tools, community involvement emerged as a strong theme throughout the conference. The issue of competing or overlapping ontologies was also raised in a workshop chaired by Winston Hide, in which those interested in gene expression and description of anatomy agreed to work together to attempt to represent this domain in a unified way. Readers who are interested in ontology developments may wish to look at the GOBO (**http://www.geneontology.org/doc/gobo.html**)

Copyright © 2003 John Wiley & Sons, Ltd.

and MGED ontology (**http://mged.sourceforge.net/ontologies/**) sites, which list existing ontologies and tools for ontology development. There are also discussion lists that run from these sites.

Three of the SOFG speakers have contributed reviews to this issue of CFG. These were selected to represent the diversity of ontology development and to illustrate the use of different ontology-building tools. Raymond Lee and Paul Sternberg describe the construction of an ontology to describe the anatomy and cell lineages of *C. elegans* [3]. Chris Stoeckert and Helen Parkinson describe the MGED ontology, which has been developed for describing microarray experiments [5], and Robert Stevens *et al.* provide an introduction to description logics for beginners, and apply the DL principles to the Gene Ontology [4].

The abstracts and the presentations from the meeting are available from **http://www.ebi.ac.uk/SOFG**.

### References

1. Brazma A, Hingamp P, Quackenbush J, *et al.* 2001. Minimum information about a microarray experiment (MIAME) — towards standards for microarray data. *Nature Genet* **29**: 365–371.
2. Karp P. 2001. Many Genbank entries for complete microbial genomes violate the Genbank standard. *Comp Funct Genom* **1**: 25–27.
3. Lee RYN, Sternberg PW. 2003. Building a cell and anatomy ontology of *C. elegans*. *Comp Funct Genom* **4**: (this issue).
4. Stevens R, Wroe C, Bechhofer S, *et al.* 2003. Building ontologies in DAML + OIL. *Comp Funct Genom* **4**: (this issue).
5. Stoeckert CJ, Parkinson H. 2003. The MGED ontology: a framework for describing functional genomics experiments. *Comp Funct Genom* **4**: (this issue).
6. The Gene Ontology Consortium. 2000. Gene ontology: tool for the unification of biology. *Nature Genet* **25**: 25–29.
7. The Gene Ontology Consortium. 2001. Creating the gene ontology resource: design and implementation. *Genome Res* **11**: 1425–1433.

Copyright © 2003 John Wiley & Sons, Ltd.
